# Metabolic Syndrome According to Dietary and Health-Related Lifestyle in Male Cancer Survivors and Non-Cancer over 40 Years of Age

**DOI:** 10.3390/foods13091351

**Published:** 2024-04-27

**Authors:** Huan Meng, Yongchul Choi, Kitae Yim

**Affiliations:** 1 Department of Physical Education, Gangneung-Wonju National University, Gangneung 25457, Republic of Korea; 20205047@gwnu.ac.kr (H.M.); ycchoi@gwnu.ac.kr (Y.C.); 2 Division of Liberal Arts, Daejin University, Pocheon 11159, Republic of Korea

**Keywords:** metabolic syndrome, cancer survivor, health-related behavior, dietary pattern

## Abstract

Researchers often report higher metabolic syndrome (MetS) pr\4;evalence among cancer survivors than among non-cancer individuals. This study aims to explore the impact of cancer presence, activity type, and dietary lifestyle on MetS in males over 40 years of age. Participants (*n* = 9846; 618 cancer survivors, 9228 non-cancer) were selected by extracting data from a Korean government database spanning the years 2016 to 2021. Physical activity patterns, dietary habits, and MetS factors were measured, and a multiple logistic regression analysis was statistically processed for an odds ratio (OR). MetS was present in 32.8% of cancer survivors and 28.6% of non-cancer individuals. Gastric cancer survivors exhibited a 16% lower OR for MetS versus non-cancer participants. The ORs were higher by 1.60-, 1.45-, and 1.26-fold for colorectal, urinary, and other cancers, respectively. Cancer survivors with high calorie, carbohydrate, and fat intakes exhibited ORs of 2.01 (95% CI 1.28−4.04), 2.33 (95% CI 1.28−4.54), and 1.39 (95% CI 1.05−2.37) compared to the recommended level. The high fiber-intake group reduced the MetS OR by 20%. In conclusion, The MetS prevalence was higher in survivors with colorectal cancer, urinary cancer, and other cancers, while it was lower in patients with gastric cancer. Survivors with low rates of eating three meals a day, high skipping breakfast, increased eating-out rate, and no nutritional learning opportunity displayed higher MetS prevalence. Additionally, cancer survivors who had more strength and leisure activities had a lower OR of MetS.

## 1. Introduction

Cancer is one of the most common diseases in Korea and has a high mortality rate. A Korean government report in 2020 revealed that the number of new cancer cases was 240,000, with 80,000 cancer-related deaths [1]. As such, Korea has a high age-standardized incidence rate of 262 per 100,000 persons, exhibiting a slightly higher incidence in men (279) than in women (257) [2].

The country’s early cancer-screening system and medical advancements have led to an increase in the detection rate and proactive implementation of preventive strategies [3]. Nonetheless, this concern persists, as the number of cancer patients has shown a consistent increase over the past two decades, with projections indicating a continuation, attributed to the aging phenomenon contributing to the expansion of the older adult population [4].

Although many persons who survive cancer regain normal functioning after treatment, some experience chronic physical and mental challenges that persist over the long term and negatively impact their quality of life [5,6]. Additionally, patients undergo severe pain and fatigue, alongside experiencing aftereffects stemming from organ resection, prolonged drug use, chemotherapy, radiation therapy, and hormonal changes [7,8]. MetS is also frequently reported among cancer survivors. An eight-year study executed in the United States exposed that the odds ratio (OR) of MetS was 2.60 times higher in cancer survivors than in non-cancer individuals [9]. A Korean researcher presented that the rate of MetS was 25.7% in cancer survivors, which was 1.56-fold higher than the 18.8% in healthy controls. Moreover, significantly higher MetS was observed in colorectal, lung, thyroid, prostate, and bladder cancer survivors [10]. Nevertheless, another report found no change in MetS in survivors but identified a 2.28-fold of having low levels of high-density lipoprotein cholesterol (HDLC), whereas in gastric cancer survivors, the OR of MetS was lower by 60% [11].

In a study on physical activity and comorbidities in cancer survivors, high blood pressure was reduced by 55% and diabetes by 36% in patients who met aerobic physical activity requirements, and fasting glucose and total cholesterol were significantly lower in the group with sufficient strength exercise [12]. Another study found that, in cancer survivors, the group with the highest physical activity had an 18% reduction in mortality before and 37% after diagnosis compared to the group with the lowest physical activity [13]. The importance of diet is also emphasized in various ways in the relationship between diet and cancer survivors. In a large-scale meta-analysis mortality study, the mortality rate was inversely proportional as vegetable and fish intake increased, and there was a proportional relationship with the amount of alcohol. A healthy dietary pattern decreased mortality by 23% after a cancer diagnosis, while a Western-style diet increased it by 1.46 times [14].

Possible causes of MetS in cancer survivors include metabolic changes [15] and low physical activity due to psychological decline, pain, and physical discomfort due to the aftereffects of treatment. As a comprehensive result, a decline in activity ensues, making it challenging to manage risk factors, including obesity, high blood pressure, and dyslipidemia [16].

However, diverse analyses and in-depth research on metabolic syndrome according to physical activity and eating style in cancer survivors are still lacking. Considering the high incidence of cancer in Korea, studies on activity and dietary lifestyle among cancer survivors are still rare. Hence, this study aims to contribute to health management by analyzing the prevalence of MetS among Korean male cancer survivors based on their dietary and health-related lifestyles.

## 2. Materials and Methods

### 2.1. Participants

Data for this study were extracted from the database of the Korean Ministry of Health and Welfare of Korea. The government’s data center is designed to understand the public health status, reflect policies, and collect data for providing to international organizations such as the World Health Organization (WHO) and the Organization for Economic Co-operation and Development (OECD).

The survey database process is as follows. To recruit participants, the country is divided into 192 regions and all age groups. Twenty-five households are selected per survey region, and all household members are eligible. An investigator visits the household to undergo testing procedures, conduct a health questionnaire, and receive prior consent. On the day of the test, the test is conducted in a special vehicle equipped with medical equipment. Various test results are uploaded to the web page as anonymized data and provided as public data for use for research purposes.

In this study, the data were selected between 2016 and 2021 with a total of 9228 non-cancer and 618 cancer cases. Five years of data were extracted from the database, and only cases that matched gender and age and had clear information on the presence or absence of cancer were included. Cases where variables had to be analyzed in various surveys for this study were omitted, and data from people who did not consent to the study were excluded. The participants in this study comprised men aged 40 years or older who had been diagnosed with cancer and those who had not been diagnosed with cancer. This is because the incidence of cancer in Korea rapidly increases after middle age [2], and the Korean government also provides free cancer screening for people over the age of 40 [17].

Other cancers accounted for less than 20 cases and include minor cancers such as skin cancer, melanoma, and blood cancer. The questionnaire assessed dietary habits, physical activity, socioeconomic status (income and education), smoking status, and drinking status. Participants filled out the written questionnaire themselves, and staff helped participants who had difficulties due to low vision or incomprehensibility of the sentences.

Prior to the assessment, the participants were instructed to fast for 8 h and were provided with a light gown and slippers. The test procedure was thoroughly explained by the physician, and written informed consent was obtained from all participants before the questionnaire was completed. Subsequently, measurements of the waistline and blood pressure were obtained, and blood samples were obtained, which are fundamental components of the MetS clinical examination. This study adhered to the ethnic research guideline of the Gangneung-Wonju National University Research Ethics Committee (approval number: 202016).

### 2.2. Health-Related Questionnaire

The socioeconomic status of the participants was assessed based on their education level and household monthly income. Household income was grouped into quintiles (Q) as monthly income according to the number of households. High is Q1 and Q2, medium is Q3, and low is Q4 and Q5. Alcohol frequency was assessed according to the weekly frequency and was categorized; low is 1 day or less, medium is 2 to 3 days, and high is 4 or more days. It was based on the alcohol risk level from the classification of the WHO [18]. The smoking classifications were ‘current’, ‘quit’, and ‘never’. Cancer survivors were asked about the type of cancer. Urinary-related cancers included prostate and bladder cancers, whereas other cancers included cancers with a low incidence, such as those of the skin, eyes, and tongue.

### 2.3. Dietary Patterns

This questionnaire was produced by the survey agency, and its reliability and validity were verified [19]. The questions required for the analysis in this study were selected. The total daily calorie intake, and the ratio of carbohydrates, fat, and protein were recorded [20]. Furthermore, the amount of fiber, number of meals per day, frequency of eating out, frequency of skipping breakfast, and nutrition education experience were investigated. The energy intake and food-consumption patterns of participants were assessed using a 24 h recall method, together with a food-frequency tool that was developed by the survey organization [21]. The questionnaire was designed to obtain information on the frequency and quantity of 112 foods. The information obtained in this way was used to compare the recommended nutrient intakes for each gender and age group. The nutrients and calories contained in the foods were calculated using a previously published method [22].

The grouping of weekly frequencies of dietary lifestyles is as follows: 3 meals a day: 4 days or more (high), 3 days or less (low); eating out: 4 or fewer meals (low), 5 to 6 meals (medium), 7 to 14 meals (high); breakfast: 5 days or more (high), 3 to 4 days (medium), 2 days or less (low). Additionally, participants were questioned regarding their experience with nutrition education [23,24].

### 2.4. Physical Activity

The questionnaire was distributed in various languages worldwide by the WHO to unify the physical activity questionnaires. Accordingly, physical activity was divided into work and leisure, and the weekly frequency of high-strength exercise was added [25,26]. The questionnaire asked participants to remember the physical activities they had performed over the past 7 days. Aerobic physical activity was categorized as moderate to hard activity, and walking, strength exercise and sedentary time were assessed. Sedentary duration encompasses the average daily time, including all non-sleeping sedentary activities, such as computer use, playing mobile games, listening to music, studying, watching television, and vehicle use. Sedentary behavior was classified into tertiles, and the recommended level of activity followed the guidelines in the literature [27].

### 2.5. MetS Diagnosis

The MetS diagnostic criteria are from the Third Report of the National Cholesterol Education Program and cover when an individual fulfills three or more of the five criteria: blood pressure: systolic 130, diastolic 85 mmHg or higher; elevated triglyceride: 150 mg/dL or higher; fasting glucose: 100 mg/dL or more; and HDLC: less than 40 mg/dL [28]. A waistline of more than 90cm was applied using Asian criteria; the thickest part of the waist, near the navel, was measured horizontally using a tape measure [29]. Being managed on prescription medication was considered a risk factor.

Blood for lipid analysis was obtained by a nurse using a needle and vacuum tube. Blood pressure was tested three times, and the mean of the 2nd and 3rd values was utilized. The waistline was measured twice, and the higher value (within an error range of 0.5 cm) was used.

### 2.6. Data Analysis

The statistical package was carried out with SPSS software (version 25.0; SPSS Inc., Chicago, IL, USA), and the normal distribution properties were verified by Shapiro–Wilk. Because the main variables examined in this study showed normal distribution, parametric analysis was adopted. The variables were presented as either mean and standard deviation or as number and percentage. An independent t-test and chi-square test were used to compare between groups, and the prevalence of MetS was derived using multiple logistic regression analysis and expressed as OR.

Through multiple regression analysis, adjustment variables to be applied in the logistic regression analysis were selected. Age, income level, smoking status, and alcohol frequency were adopted as adjustment variables. The prevalence analysis according to dietary habits and nutrients included only cases of colorectal, urinary, and other cancers that showed a significant increase in MetS, and the reference group was a healthy group without cancer and MetS. Statistical significance (*p*) was established at <0.05 with a 95% confidence interval (CI).

## 3. Results

### 3.1. General Information Profile

MetS occurred in 28.6% of cancer patients and 32.8% of non-cancer individuals (Table 1). The MetS group showed significant differences compared to the non-MetS (NMetS) group in terms of weight, body mass index (BMI), waistline, systolic and diastolic blood pressure, HDLC, triglyceride, glucose, household income, and alcohol frequency, with height being excluded.

Household income was significant depending on the presence or absence of cancer and MetS. Alcohol frequency was associated with MetS in both the non-cancer and cancer-survivor groups. Within each NMetS and MetS group, comparisons were made according to cancer or non-cancer. In both the NMetS and MetS groups, diastolic blood pressure and triglyceride were higher in cancer than in non-cancer.

### 3.2. Metabolic Syndrome and Risk Factors for Various Cancers

Through multiple regression analysis, the adjustment variables were derived for logistic regression analysis. It was age, income level, smoking status, and alcohol frequency (*p* < 0.05).

**Figure 1:**
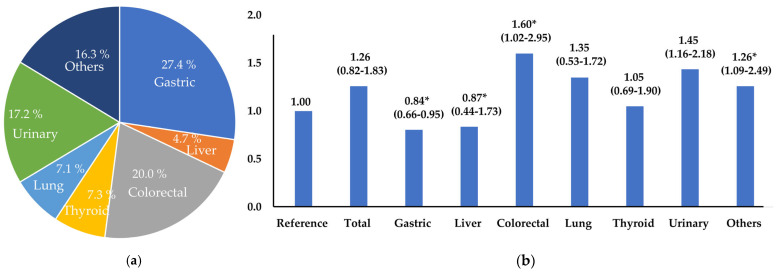
(**a**) Ratio of cancer type and (**b**) metabolic syndrome odds ratio of cancer type. * *p* < 0.05, significance level; Two cancer, 36 patients; Three cancer, 2 patients. Reference group: non–cancer with non–metabolic syndrome (1.00); Adjusted variables included age, income, smoking, and alcohol status.

The non-cancer group with NMetS was set as the reference group (1.00) and applied to calculate the OR of MetS for cancers (Figure 1). The OR for the presence of MetS regarding overall cancer survival was not significant. However, the presence of MetS was higher, at 1.60-, 1.45-, and 1.26-fold, in patients with colorectal, urinary, and other cancers, respectively. No significant alteration in MetS risk was noted among liver and lung cancer survivors. However, notably, gastric cancer survivors demonstrated a 16% lower MetS risk compared to the reference group.

### 3.3. MetS Prevalence According to Nutritional Intake and Dietary Habits

In non-cancer patients, high-calorie intake had a higher MetS prevalence, by 1.8-fold, and the high-carbohydrate group showed a 1.96-fold higher prevalence. The low-calorie intake group exhibited a 12% reduction in the MetS and the carbohydrate groups lower than the RDI had a 17% lower. The fat, protein, and dietary fiber levels were not significantly different.

Among cancer survivors, there were higher 2.01-, 2.33-, and 1.39-fold increases in the MetS prevalence in the high-calorie, high-carbohydrate, and high-fat groups, respectively. The OR for protein intake was non-significant, but MetS prevalence was 20% lower in the group consuming a large amount of dietary fiber (Table 2).

**Figure 2:**
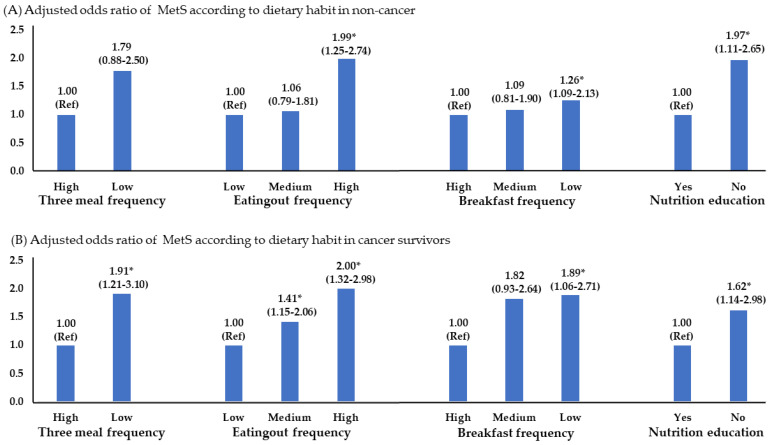
Adjusted odds ratio of MetS in dietary lifestyle. * *p* < 0.05, significance level. Adjusted variables: age, income level, smoking, and alcohol status.

Consuming three meals did not impact the risk of MetS in non-cancer, but eating out frequently increased it by 1.99 times. There was a 1.26-fold increase in those who consumed breakfast infrequently. Among cancer survivors, eating less than three times per day saw the MetS prevalence increase by 1.9-fold, with a 2-fold increase observed with high eating out, and 1.89-fold in the breakfast skipping group. Further, a 1.6-fold increase in the MetS prevalence was observed in participants who did not receive nutritional education (Figure 2).

### 3.4. MetS Prevalence According to Physical Activity Pattern

**Figure 3:**
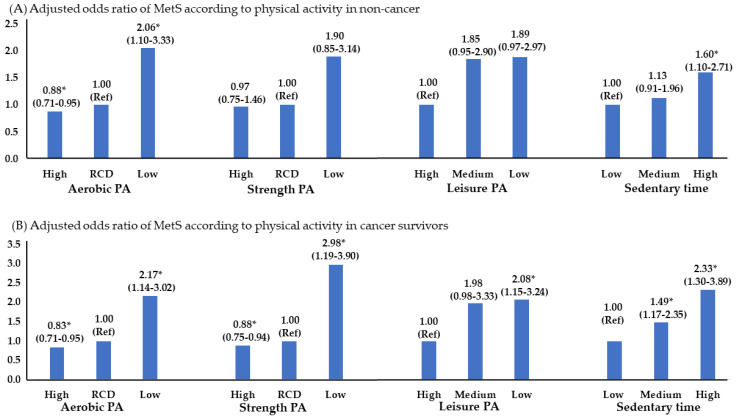
Adjusted odds ratio of MetS in physical activity pattern. * *p* < 0.05, significance level. PA, physical activity; RCD, recommendation; adjusted variables: age, income level, smoking, and alcohol status.

Deceased aerobic PA was higher by 2.06-fold and 2.17-fold in both non-cancer and cancer survivors below the recommended level, and strength exercise below the recommended level was higher by 2.98-fold only in the cancer-survivor group. It was 1.60 times in the non-cancer group with a sedentary lifestyle, and 2.33 times in the cancer-survivor group (Figure 3). Meanwhile, non-cancer individuals displayed no significant differences in strength exercise and leisure activities, while the cancer-survivor group exhibited significant values across all parts of physical activity.

## 4. Discussion

The well-being of cancer survivors encompasses their mental and physical health, as well as their ability to recover from the aftereffects of cancer treatment. This study investigated the metabolic health status of Korean male cancer survivors taking into account their physical activity levels, dietary patterns, and nutritional intakes.

This study revealed that MetS occurred in 32.8% of cancer survivors and 28.6% of non-cancer individuals. Although there was no overall change in the OR for MetS in all cancer survivors, the outcomes differed based on the cancers. The MetS risk was relatively low in gastric cancer survivors but increased in those with colorectal, urinary tract, and other cancers. This aligns with the results reported in a prior study. The study found that the prevalence of MetS was not significant when analyzed by cancer type, as well as among overall cancer survivors. However, it observed a 58% decrease in MetS among stomach cancer patients [11].

Similarly, in 12,734 male cancer survivors aged ≥40 years in the United States, the MetS prevalence was higher by 2.18 times than healthy individuals. In particular, the MetS prevalence was 2.85-, 2.87-, and 4.88-fold for prostate, colorectal, and blood cancers, respectively. No significant differences in melanoma, bladder, or other cancers were identified [9]. Patients with gastric cancer often experience significant weight loss due to poor digestion and low absorption rates, as well as small meal sizes owing to gastrectomy [30]. The findings of the current study are thought to be influenced by comparable factors. To prevent underweight and sarcopenia, individually tailored exercise and diet therapy plans need to be developed.

The mechanism underlying the development of MetS that occurs after cancer treatment may be related to a lack of physical activity and changes in dietary patterns as aftereffects of cancer treatment. Additionally, increased levels of stress hormones such as cortisol may negatively affect insulin sensitivity. Moreover, chemotherapy or hormone therapy may induce alterations in body composition, and there is a possibility of increased insulin resistance due to an increase in inflammatory cells [31,32]. By far, the most well-established approach is that dietary and lifestyle modifications are the most probable and effective methods for reducing the risk of MetS [33]. The development of MetS in survivors is undoubtedly influenced by physical activity and diet and requires close monitoring; hence, the habitual patterns in survivors need to be investigated.

**Figure 4:**
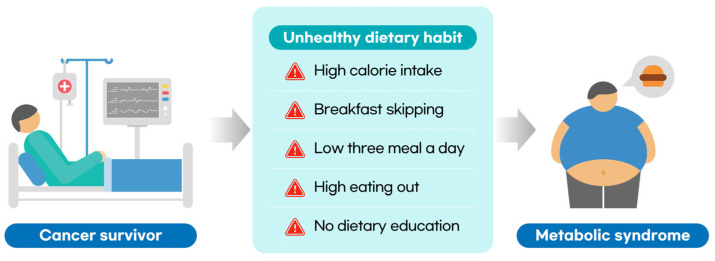
The relationship between metabolic syndrome and dietary habit in cancer.

In this analysis, the calorie intake and carbohydrate intake rate in cancer survivors showed the same results as non-cancers (Figure 4). Meanwhile, other results of cancer survivors showed that MetS was significantly higher in the high-fat and low-fiber consuming groups. In particular, fiber, which is mainly present in fruits and vegetables, has been shown to aid in digestion and in preventing constipation [34]. A meta-analysis of 11 studies revealed that fiber reduced the odds ratio for MetS by 30%. However, the results of the analysis of three cohorts in the same study were not statistically significant [35]. The primary mechanisms by which dietary fiber exerts its effects are through its impact on obesity and insulin resistance. Dietary fiber plays a crucial role in reducing nutrient absorption, suppressing, and appetite, and regulating energy homeostasis. In addition, it decreases the glycemic index of food, improves glucose homeostasis, regulates inflammatory cytokines, and alters the intestinal microbiome [35,36]. However, researchers should be aware of research results showing that fiber consumption does not significantly change metabolic syndrome [34,37].

High-calorie diets, particularly high-carbohydrate and high-fat diets, negatively affect metabolic health. The accumulation of excess calories leads to an increase in body fat and abdominal obesity. Moreover, insulin resistance increases, which prevents insulin from exerting its full impact, and increases TG levels. As a result, it has been suggested that carbohydrate intake should be managed so as not to exceed 55% of the total energy intake [38,39]. A study analyzing a decade-long dataset from the United States contradicts the outcomes observed in this investigation. Employing the recommended caloric intake as a reference, this study revealed a 1.067-fold elevation in MetS incidence among individuals consuming fewer calories than recommended—a disparity from the findings of the present study. Nonetheless, deleterious implications were associated with fat consumption. A positive relation was observed between heightened fat intake and a 1.271-fold increase in MetS risk [40]. Thus, the findings underscore the imperative of reducing fat consumption concomitant with judicious carbohydrate intake, as articulated within this study.

In general, dietary guidelines emphasize the importance of having breakfast, eating three times per day, and eating out less frequently. Research has shown that individuals who skipped breakfast, ate three meals less frequently, and ate out frequently exhibit a higher risk of developing MetS. Dietary specialists emphasize the significance of breakfast due to its impact on ghrelin and leptin, which are involved in regulating metabolism and appetite [41]. Although skipping breakfast temporarily lowers blood-sugar levels due to fasting, this causes insulin resistance, which makes cells less sensitive to insulin and ultimately increases blood-sugar levels. Furthermore, forgoing breakfast might elevate the probability of consuming calorie-dense foods later in the day, potentially leading to overeating and contributing to obesity [42,43].

Therefore, a nutritious breakfast can help reduce total calories, regulate hormonal homeostasis, and control appetite [44]. Occasionally, there are conflicting reports suggesting that skipping meals, like intermittent fasting, aids in weight management, with some asserting that skipping breakfast specifically helps reduce the risk of obesity [45,46]. Furthermore, another study reported that skipping breakfast had no effect on insulin sensitivity or ghrelin or leptin levels [47].

Perceptions regarding eating out may vary, but generally, eating out tends to be viewed unfavorably because of the high calorie, salt, and sugar contents of meals [48]. In this study, the frequency of MetS was higher 1.11 to 2.65 times in the group that ate out frequently compared to those who ate out less frequently [49]. Experts recommend curtailing the frequency of eating out as much as possible and advise that more attention should be paid to controlling dietary cravings and consuming balanced nutrition when eating out [50].

The current study uncovered that insufficient physical activity levels raised the likelihood of developing MetS. This applies not only to aerobics but also to strength exercise and leisure activities. The rationale for emphasizing physical activity in cancer survivors is multifaceted. Physical activity has a favorable impact not only on the risk of MetS, but also on obesity, depression, fatigue, quality of life, and cancer recurrence rates [51,52]. Research conducted with breast cancer survivors demonstrated that engaging in exercise enhanced insulin-like growth factor I, and an analysis of studies in several cancers found that physical activity lowered BMI, weight, aerobic fitness, muscle power, and walking capacity, and greatly improved the overall quality of life of patients [53]. In addition, a large meta-analysis found that survivors with higher physical activity had an 18% lower mortality rate from cancer and a 37–42% lower likelihood of developing breast and colorectal cancers [13].

The current study underscores the intricate interplay between cancer and MetS and suggests that specific mechanisms may be contingent upon the type of cancer and individual circumstances. MetS management for cancer patients often requires a multidisciplinary approach that addresses both cancer and the metabolic health of patients through appropriate medical interventions, lifestyle modifications, and supportive care. Optimizing the overall well-being of patients with cancer requires regular monitoring and collaboration between oncologists and other healthcare providers. The results of this study may be significant in that they emphasize the need to have a multifaceted perspective regarding these health-related factors.

There are certain limitations to this study. Given the inherent nature of cross-sectional research, establishing causal relationships is difficult. This is because participants may already have had MetS before developing cancer. Certain studies have indicated a heightened occurrence of cancer in individuals with MetS. However, conducting such research is challenging. Patients with cancer require long-term follow-up, and the dropout rate is high. Due to the difficulty of regional travel, the hospitals where surgery was performed and the hospitals that provided long-term care often did not match. In addition, health behaviors such as those associated with MetS are also highly likely to occur in clusters. For example, it is possible that groups with low physical activity levels may also have unhealthy dietary habits. Although this study independently analyzed the factors, additional research is required to analyze the complex health behaviors and causes of MetS. Notably, the finding of this analysis showed a significant increase in MetS prevalence only in colorectal, urinary tract, and other cancers. However, further research regarding the underlying biological mechanisms is still needed. In addition, future research should include a broad range of issues, for example, frequent snacking, late-night snacks, irregular meals and binge eating, eating alone, one food diet, and eating more than four meals a day. In particular, the recommended guideline diet for cancer patients is low in red meat, sodium, and carbohydrates and high in fruits and vegetables, and dietary fiber [54]. Therefore, research on their long-term effects must still continue, and in-depth research will not only expand the field but also help actual cancer patients.

## 5. Conclusions

The MetS prevalence was 1.26 to 1.6 times higher in individuals with colorectal cancer, urinary cancer, and other cancers, while it was lower in patients with gastric cancer. They consumed a diet with high calories, and high percentages of carbohydrates and fat, and displayed low-strength exercise and leisure activity. Additionally, skipping breakfast and eating out more increased the prevalence of MetS. Therefore, a personalized health management system, such as nutritional education and consultation with physical activity experts, may be required to systematically manage the metabolic health of colorectal, urinary, and other cancer survivors.

## Figures and Tables

**Table 1:** General information profile of non-cancer and cancer survivors.

Variables	Non-Cancer (*n* = 9228)		*p*	Cancer Survivors (*n* = 618)		*p*	Non-Cancer vs. Cancer Survivors	
	NMetS (*n* = 6593, 71.4%)	MetS (*n* = 2635, 28.6%)		NMetS (*n* = 415, 67.2%)	MetS (*n* = 203, 32.8%)		within NMetS	within MetS
Age, years	58.4 ± 11.7	61.7 ± 11.1	<0.001	67.0 ± 9.5	68.7 ± 9.2	0.039	<0.001	<0.001
Height, cm	168.0 ± 6.3	167.9 ± 5.9	0.458	167.5 ± 6.3	168.5 ± 6.3	0.108	0.158	0.068
Weight, kg	66.7 ± 9.5	71.8 ± 9.1	<0.001	65.2 ± 8.6	75.1 ± 10.5	<0.001	0.203	<0.001
BMI, kg/m^2^	23.6 ± 2.7	25.4 ± 2.6	<0.001	23.2 ± 2.6	26.3 ± 2.9	<0.001	<0.001	<0.001
Waistline, cm	84.3 ± 7.4	92.8 ± 7.3	<0.001	83.9 ± 7.4	93.6 ± 7.3	<0.001	0.250	0.029
SBP, mmHg	121.1 ± 16.9	129.3 ± 15.2	<0.001	120.4 ± 15.0	128.2 ± 15.2	0.031	0.356	0.321
DBP, mmHg	73.0 ± 9.6	74.8 ± 10.8	<0.001	76.8 ± 9.8	79.1 ± 11.5	<0.001	<0.001	<0.001
HDLC, mg/dL	50.0 ± 12.0	38.8 ± 11.0	<0.001	49.0 ± 11.1	35.1 ± 10.4	<0.001	0.066	0.034
TG, mg/dL	118.1 ± 77.2	157.5 ± 95.5	<0.001	146.8 ± 93.5	195.9 ± 153.0	<0.001	<0.001	<0.001
Glucose, mg/dL	103.5 ± 24.3	116.9 ± 28.9	<0.001	101.8 ± 23.2	119.6 ± 31.6	<0.001	0.162	0.233
Income level								
High	2321 (35.2%)	648 (24.6%)	0.015	188 (45.4%)	54 (26.4%)	<0.001	<0.001	0.008
Medium	2215 (33.6%)	925 (35.1%)		130 (31.3%)	62 (30.3%)			
Low	2057 (31.2%)	1062 (40.3%)		97 (23.3%)	88 (43.3%)			
Education level								
To middle school	1464 (22.2%)	606 (23.0%)	0.273	61 (14.7%)	54 (26.4%)	0.014	<0.001	0.098
To high school	3013 (45.7%)	1241 (47.1%)		200 (48.3%)	102 (50.3%)			
Above college	2116 (32.1%)	788 (29.9%)		154 (37.0%)	47 (23.3%)			
Smoking statue								
Present smoker	3382 (51.3%)	1233 (46.8%)	0.207	211 (50.9%)	109 (53.6%)	0.373	0.128	0.446
Quitting smoking	1464 (22.2%)	593 (22.5%)		76 (18.3%)	35 (17.1%)			
No experience	1747 (26.5%)	809 (30.7%)		128 (30.8%)	59 (29.3%)			
Alcohol frequency								
Low	3910 (59.3%)	1391 (52.8%)	<0.001	374(64.1%)	247(58.8%)	<0.001	0.367	0.373
Medium	1563 (23.7%)	698 (26.5%)		134(22.9%)	103(24.5%)			
High	1121 (17.0%)	545 (20.7%)		76(13.0%)	70(16.7%)			

NMetS, non-metabolic syndrome; MetS, metabolic syndrome; BMI, body mass index; SBP, systolic blood pressure; DBP, diastolic blood pressure; HDLC, high-density lipoprotein cholesterol; TG, triglyceride.

**Table 2:** Metabolic syndrome odds ratio and nutrition consumption.

Variables	Class	Metabolic Syndrome Odds Ratio (95% Confidence Interval)	
		Non-Cancer	Cancer Survivors
Calorie intake	kcal/day		
Low	<2000	0.88 (0.60–0.99)	0.82 (0.60–0.95)
RDI	2000–2400	1.00	1.00
High	>2400	1.84 (1.13–3.28)	2.01 (1.28–4.04)
Carbohydrate	%		
Low	<55	0.83 (0.61–0.95)	0.84 (0.54–0.98)
AMDR	55–65	1.00	1.00
High	>65	1.96 (1.08–3.67)	2.33 (1.28–4.54)
Fat	%		
Low	<15	0.83 (0.96–1.82)	0.86 (0.98–1.95)
AMDR	15–30	1.00	1.00
High	> 30	1.23 (0.76–3.33)	1.39 (1.05–2.37)
Protein	%		
Low	<7	0.80 (0.62–2.27)	0.72 (0.54–2.17)
AMDR	7–20	1.00	1.00
High	>20	1.26 (0.71–2.58)	1.61 (0.45–4.12)
Dietary fiber	g/day		
Low	<20	1.99 (0.76–3.01)	1.75 (1.09–3.12)
RDI	20–25	1.00	1.00
High	>25	0.78 (0.58–1.97)	0.80 (0.69–0.93)

RDI, recommended dietary intakes; AMDR, acceptable macronutrient distribution range; adjusted variables: age, income, smoking, and alcohol status.

## Data Availability

The data in this study are publicly available data distributed by government agencies: [https://knhanes.kdca.go.kr, accessed on 13 January 2024].
